# Mechanism and Application Prospects of NLRC3 Regulating cGAS-STING Pathway in Lung Cancer Immunotherapy

**DOI:** 10.7150/ijms.102328

**Published:** 2024-10-07

**Authors:** Qichao Wang, Zhen Ren, Jianing Zhao, Tianliang Zheng, Lifei Tong, Jing Liu, Zhaoxia Dai, Shuhong Tang

**Affiliations:** 1 Dalian Medical University, Dalian 116044, Liaoning, China.; 2 Department of Medical Oncology, The Fifth People's Hospital of Dalian, Dalian 116021, Liaoning, China.; 3 Faculty of Medicine, Dalian University of Technology, Dalian 116024, Liaoning, China.; 4 Central Hospital of Dalian University of Technology, Dalian 116003, Liaoning, China.; 5 Department of Radiotherapy, The Fifth People's Hospital of Dalian, Dalian 116021, Liaoning, China.; 6 Department of Thoracic Oncology, The Second Affiliated Hospital of Dalian Medical University, Dalian 116021, Liaoning, China.

## Abstract

NLRC3, a negative regulator, exhibits considerable potential in the realm of lung cancer immunotherapy by virtue of its profound impact on the immune response intensity, primarily through its regulatory effects on the cGAS-STING pathway. The inhibition of NLRC3 has been found to augment the activity of the aforementioned pathway, thereby enhancing the anti-tumor immune response. This comprehensive review endeavors to elucidate the molecular and genetic structures of NLRC3, its role within the immune system, and its interaction with the cGAS-STING pathway, with a particular emphasis on its potential applications in lung cancer immunotherapy. Existing research underscores NLRC3's capacity to mitigate excessive immune responses via the negative regulation of the cGAS-STING pathway, thus underscoring its significant regulatory role in lung cancer immunotherapy. The development of pharmaceutical interventions and gene therapy strategies targeting NLRC3 presents a promising avenue for the creation of novel therapeutic options for individuals afflicted with lung cancer. Nonetheless, the clinical application of these therapies is confronted with both technical and biological challenges. This review aims to provide a theoretical foundation for related research endeavors and delineate future research directions in this field.

## Background

Lung cancer is a paramount global health concern, accounting for a substantial proportion of cancer-related incidence and mortality worldwide, with a staggering number of new cases and fatalities reported annually. According to the World Health Organization, lung cancer is responsible for over 18% of all cancer-related deaths, thereby presenting a formidable challenge to global public health 1-3. Despite the advent of novel diagnostic techniques and therapeutic interventions, overall survival rates remain disappointingly low, particularly among patients with advanced lung cancer 4, 5. The inherent heterogeneity of tumors, the intricate molecular mechanisms governing their behavior, and the emergence of resistance to existing treatments are pivotal factors contributing to the suboptimal outcomes of lung cancer therapy 6, 7.

Immunotherapy, a nascent yet promising cancer treatment modality, leverages the patient's immune system to recognize and target cancer cells, offering a higher degree of specificity and more durable effects compared to traditional therapeutic approaches such as surgery, radiation, and chemotherapy 8, 9. The deployment of immune checkpoint inhibitors (e.g., PD-1/PD-L1 inhibitors) in non-small cell lung cancer (NSCLC) has yielded significant improvements in survival rates among certain patients 8, 10. Nonetheless, challenges persist in the application of immunotherapy for lung cancer, including resistance in some patients and the intricate complexity of the tumor microenvironment 6-8.

The cGAS-STING pathway is a vital innate immune signaling pathway that detects cytosolic DNA and activates downstream signaling cascades, resulting in the production of type I interferons and other inflammatory cytokines, which initiate anti-tumor immune responses 10-12. Recent studies have underscored the critical role of the cGAS-STING pathway in anti-tumor immunity, particularly in lung cancer, where its activation significantly enhances immune activity within the tumor microenvironment, thereby suppressing tumor growth and metastasis 8, 13. NLRC3 (NLR family CARD domain-containing protein 3), a negative regulator, has garnered considerable attention in recent years for its role in modulating the cGAS-STING pathway 14-16. Studies have demonstrated that NLRC3 modulates immune response intensity by inhibiting the cGAS-STING pathway, playing a pivotal role in the immune system 17-19. Consequently, exploring the structure and function of NLRC3, analyzing its regulatory mechanisms within the cGAS-STING pathway, and assessing its potential in lung cancer immunotherapy are of paramount significance 20, 21.

## Advances in the Study of NLRC3 Structure and Function

NLRC3 (NLR family CARD domain-containing protein 3) is a member of the NOD-like receptor (NLR) family, characterized by its unique molecular structure comprising an N-terminal nucleotide-binding oligomerization domain (NOD), a C-terminal leucine-rich repeat (LRR) domain, and a central nucleotide-binding domain (NACHT) 22, 23. This distinctive structure confers NLRC3 with significant regulatory functions in immune responses 24-26. The NLRC3 gene is situated on human chromosome 16q13 and consists of multiple exons and introns 27, 28. Gene transcription products undergo splicing to form mRNA, which is subsequently translated into functional proteins 29-31. Gene expression exhibits considerable variability across different cell types and physiological conditions 32, 33. The NOD domain of the NLRC3 protein is responsible for ATP binding and hydrolysis, which is central to its functional activity 34-36. The LRR domain primarily mediates protein-protein interactions, including the recognition and binding of specific ligands or proteins 37, 38. The NACHT domain regulates by promoting protein oligomerization and activating downstream signaling pathways 39-41.

NLRC3 acts as a negative regulator in the innate immune system, modulating immune response intensity by inhibiting key signaling pathways such as cGAS-STING. Its primary functions include inhibiting cGAS-STING-mediated type I interferon production to prevent excessive inflammatory responses, regulating the activity of other NOD-like receptors to maintain immune system balance, and further modulating immune responses through interactions with other immune regulatory proteins 42, 43. Specifically, NLRC3 interacts directly with STING to prevent its oligomerization and downstream signaling, thus inhibiting the activation of the cGAS-STING pathway 44, 45.

NLRC3 exhibits widespread expression across various tissues and cell types, including immune cells (e.g., macrophages and dendritic cells), epithelial cells, and certain tumor cells. Its expression levels are regulated by cell type, pathological state, and external stimuli. NLRC3 is involved not only in tumor immunity but also in autoimmune diseases, infectious diseases, and metabolic disorders 46, 47. In autoimmune diseases such as systemic lupus erythematosus (SLE) and rheumatoid arthritis (RA), NLRC3 influences disease progression by regulating inflammatory responses and immune cell activity. In infectious diseases, NLRC3 protects the host from excessive inflammatory damage by modulating pathogen-induced immune responses 48-50. Furthermore, NLRC3's role in metabolic disorders such as obesity and type 2 diabetes is gaining recognition, as it influences disease progression by regulating metabolic pathways and inflammatory responses. Further research into the molecular mechanisms and regulatory networks of NLRC3 may facilitate the development of novel therapeutic strategies, offering fresh insights into the treatment of various diseases 51, 52.

## Mechanisms and Recent Advances of the cGAS-STING Pathway

The elucidation of the cyclic GMP-AMP synthase-stimulator of interferon genes (cGAS-STING) pathway represents a paradigm shift in our understanding of innate immunity. The identification of cGAS in 2013 as a cytosolic DNA sensor unveiled a critical mechanism by which cells detect and respond to aberrant intracellular DNA, initiating immune responses essential for combating pathogens and tumorigenesis. This intricate pathway, with cGAS and STING as its core components, forms a fundamental axis of intracellular innate immune signaling and host defense 53, 54.

Functionally, cGAS, residing within the cytoplasm, recognizes and binds double-stranded DNA (dsDNA) through its C-terminal DNA-binding domain. This interaction activates the enzymatic activity of cGAS, catalyzing the synthesis of cyclic GMP-AMP (cGAMP) from GTP and ATP. Acting as a crucial second messenger, cGAMP binds to and activates STING on the endoplasmic reticulum membrane. This binding triggers a conformational change in STING, facilitating its translocation to the Golgi apparatus 55, where it initiates downstream signaling cascades. These cascades culminate in the phosphorylation of TANK-binding kinase 1 (TBK1), a crucial kinase regulating antiviral and other immune responses, and interferon regulatory factor 3 (IRF3), a pivotal transcription factor governing interferon expression and antiviral immunity 11, 14. Ultimately, these activations converge to induce the production of type I interferons, such as IFN-β, which are indispensable for antiviral defense, mitigating cellular damage, and orchestrating robust host immune responses 8, 9, 56. Concurrently, the STING pathway, through NF-κB signaling, drives the expression of inflammatory cytokines, including TNF-α and IL-6, thereby modulating both local and systemic inflammation 57, 58. These inflammatory factors are critical for amplifying antiviral defenses by regulating immune cell activation and trafficking, ultimately enhancing immune responses.

Beyond its role in pathogen defense, the cGAS-STING pathway exhibits pleiotropic effects, including the regulation of cellular stress responses, clearance of damaged cells, and maintenance of immune homeostasis, thereby establishing a robust defense against viral and tumor challenges 58-60. The ability of this pathway to recognize intracellular DNA and activate downstream signaling underscores its pivotal role in innate immunity and provides novel insights into the pathogenesis of diverse diseases 61, 62.

In recent years, the role of the cGAS-STING pathway in cancer, particularly within the tumor microenvironment, has garnered significant attention. This pathway exhibits a paradoxical duality, capable of suppressing tumor growth by eliciting anti-tumor immune responses, yet simultaneously potentially promoting tumor progression through chronic inflammation. In lung cancer, for example, activation of the cGAS-STING pathway exerts a profound influence on the tumor microenvironment. Specifically, this activation enhances antigen presentation and CD8+ T cell function, augmenting anti-tumor immune responses and facilitating the elimination of tumor cells 8, 11, 13, 14. However, sustained activation of this pathway may paradoxically allow tumor cells to adapt to immune surveillance, thereby promoting tumor growth and immune evasion 9, 10. This adaptation can manifest as an upregulation of anti-inflammatory factors by lung cancer cells to suppress surrounding immune cells, effectively maintaining an immunosuppressive tumor microenvironment while exploiting STING-driven inflammation to enhance invasiveness and metastasis 21, 59. Consequently, the cGAS-STING pathway assumes a complex and critical role in lung cancer progression and immune evasion, necessitating further investigation to fully elucidate its therapeutic potential and address the challenges associated with targeting it for lung cancer therapy.

Preclinical studies have demonstrated that activating the cGAS-STING pathway can significantly enhance the response of lung cancer patients to immunotherapy. For instance, administering cGAMP or other STING agonists can amplify immune activity within the tumor microenvironment, promoting the maturation and function of antigen-presenting cells and bolstering effector T cell activity 60-62. Furthermore, combining these approaches with immune checkpoint inhibitors, such as PD-1/PD-L1 inhibitors, can overcome certain forms of immunotherapy resistance, leading to substantial improvements in treatment outcomes 63-65. However, sustained STING activation poses the risk of chronic inflammation and tissue damage, potentially fueling tumor progression and immune escape. This detrimental effect may involve excessive activation of STING-mediated NF-κB signaling, resulting in the production of inflammatory factors that can foster tumorigenesis within the tumor microenvironment 66. Tumor cells may also circumvent cGAS-STING surveillance by upregulating negative regulators like NLRC3 or downregulating STING expression 67, 68.

Therapeutic strategies targeting the cGAS-STING pathway are currently undergoing intensive investigation. Small-molecule STING agonists and cGAMP analogs have demonstrated promising anti-tumor effects in preclinical studies and are now progressing into clinical trials to assess their safety and efficacy in human subjects 69, 70. Additionally, gene-editing technologies, such as CRISPR-Cas9, are being harnessed to precisely regulate the cGAS-STING pathway, aiming to enhance the specificity and efficacy of anti-tumor immune responses 71, 72. Furthermore, studies are exploring the synergistic potential of combining cGAS-STING pathway activation with other treatment modalities, such as radiotherapy and chemotherapy, to achieve superior overall treatment outcomes 73-75.

In summary, significant strides have been made in unraveling the intricacies of the cGAS-STING pathway in lung cancer, providing a critical theoretical foundation for the development of novel therapeutic strategies. Despite the inherent challenges, targeting the cGAS-STING pathway holds immense therapeutic promise. Future research endeavors will delve deeper into the regulatory mechanisms governing this pathway, optimize existing treatment regimens, and develop more effective combination therapies to improve patient outcomes and overall prognosis 76-78.

The discovery of the cGAS-STING pathway (cyclic GMP-AMP synthase-stimulator of interferon genes) represents a major milestone in the field of innate immunity. In 2013, cGAS was first identified as a cytosolic DNA sensor, revealing how cells recognize abnormal intracellular DNA and activate immune responses to combat pathogens and tumors. This mechanism is a core component of intracellular innate immune signaling, playing a crucial role in host defense. The cGAS-STING pathway comprises cGAS and STING. cGAS, located in the cytoplasm, recognizes and binds double-stranded DNA (dsDNA) via its C-terminal DNA-binding domain, which activates its enzymatic activity to catalyze the synthesis of cyclic GMP-AMP (cGAMP) from GTP and ATP.cGAMP, as a second messenger, binds and activates STING on the endoplasmic reticulum membrane, inducing a conformational change and translocation to the Golgi apparatus, where it activates downstream signaling cascades, including the phosphorylation of TBK1 (TANK-binding kinase 1) and IRF3 (interferon regulatory factor 3) 11, 14. TBK1 is a key kinase that regulates antiviral responses and other immune reactions 13. IRF3 is a crucial transcription factor responsible for regulating interferon expression and plays a central role in antiviral immune responses. These activations ultimately lead to the production of type I interferons (e.g., IFN-β), which are essential in antiviral defense, preventing cellular damage, and activating the host immune system 8, 9. Additionally, the STING pathway induces the expression of inflammatory cytokines, such as TNF-α and IL-6, through NF-κB signaling, thereby regulating local and systemic inflammation. These inflammatory factors are crucial in antiviral defense by modulating immune cell activation and migration, thereby enhancing immune responses 10, 21. The cGAS-STING pathway also plays a key role in regulating cellular stress responses, clearing damaged cells, and maintaining immune homeostasis, forming a robust barrier against viral and tumor challenges 59, 60. By recognizing intracellular DNA and activating downstream signaling, the cGAS-STING pathway is pivotal in innate immunity, providing new insights into the pathogenesis of various diseases 61, 62.

Recently, the role of the cGAS-STING pathway in cancer has garnered significant attention. This pathway has a dual role in the tumor microenvironment: it can suppress tumor growth by inducing anti-tumor immune responses, but it may also promote tumor progression through chronic inflammation. In lung cancer, activation of the cGAS-STING pathway significantly influences the tumor microenvironment. Specifically, cGAS-STING pathway activation enhances antigen presentation and CD8+ T cell function, boosting anti-tumor immune responses and aiding in the elimination of tumor cells 8, 11, 13, 14. However, sustained activation of this pathway may allow tumor cells to adapt to immune surveillance, promoting tumor growth and immune evasion 9, 10. Some lung cancer cells upregulate anti-inflammatory factors to suppress surrounding immune cells, maintaining an immunosuppressive tumor microenvironment while exploiting STING-driven inflammation to enhance invasiveness and metastasis 21, 59. Therefore, the cGAS-STING pathway plays a complex and critical role in lung cancer progression and immune evasion. Further research is needed to explore the potential and challenges of targeting the cGAS-STING pathway for lung cancer therapy. Preclinical studies have shown that cGAS-STING pathway activation can significantly enhance lung cancer patients' response to immunotherapy. For example, cGAMP or other STING agonists can enhance immune activity in the tumor microenvironment, promoting the maturation and function of antigen-presenting cells and boosting effector T cell activity 60-62.

Additionally, combining these approaches with immune checkpoint inhibitors (e.g., PD-1/PD-L1 inhibitors) can overcome some forms of immunotherapy resistance, significantly improving treatment outcomes 63-65. However, sustained STING activation may cause chronic inflammation and tissue damage, potentially promoting tumor progression and immune escape. This may involve excessive activation of STING-mediated NF-κB signaling, resulting in the production of inflammatory factors that may promote tumorigenesis in the tumor microenvironment 66. Tumor cells may also escape cGAS-STING surveillance by upregulating negative regulators like NLRC3 or downregulating STING expression 67, 68. Therapeutic strategies targeting the cGAS-STING pathway are currently under active investigation. Small-molecule STING agonists and cGAMP analogs have shown promising anti-tumor effects in preclinical studies and are now in clinical trials to assess their safety and efficacy 69, 70. Gene-editing technologies like CRISPR-Cas9 are also being used to precisely regulate the cGAS-STING pathway, aiming to improve the specificity and efficacy of anti-tumor immune responses 71, 72. Studies are exploring the combination of cGAS-STING pathway activation with other modalities, such as radiotherapy and chemotherapy, to enhance overall treatment outcomes 73-75. In summary, significant progress has been made in understanding the cGAS-STING pathway in lung cancer, offering critical theoretical support for the development of new therapeutic strategies. Despite the challenges, targeting the cGAS-STING pathway holds significant therapeutic potential. Future research will further investigate the regulatory mechanisms of this pathway, optimize existing treatment regimens, and develop more effective combination therapies to improve patient outcomes and prognosis 76-78.

## 3. Interaction between NLRC3 and cGAS-STING pathway

Recent research has focused on the intricate interplay between NLRC3 and the cGAS-STING pathway, revealing that NLRC3 functions as a critical negative regulator, fine-tuning the cGAS-STING pathway through multiple mechanisms to maintain immune homeostasis. Firstly, NLRC3 directly interacts with STING, hindering its oligomerization and intracellular trafficking, thereby attenuating the activation of downstream signaling cascades 14, 44. Additionally, NLRC3 binds to TBK1, interfering with its activation and downstream function, ultimately inhibiting IRF3 phosphorylation and the production of type I interferons 51. Through these mechanisms, NLRC3 effectively downregulates the cGAS-STING pathway, preventing excessive and potentially detrimental immune and inflammatory responses. Furthermore, NLRC3 can directly inhibit cGAS activation by competing for DNA binding sites, thereby reducing cGAMP production 10, 14. By binding to STING and preventing its translocation from the endoplasmic reticulum to the Golgi apparatus, NLRC3 effectively inhibits downstream signaling events 21, 44.

NLRC3 exerts its regulatory influence over the cGAS-STING pathway at multiple levels, exemplified by its ability to bind to TBK1, inhibiting its activation and subsequently suppressing the phosphorylation of IRF3, as well as the production of type I interferons and inflammatory cytokines 51, 79. These diverse mechanisms position NLRC3 as a crucial modulator of the immune system, preventing tissue damage and chronic inflammation that can arise from excessive immune responses 64, 79, 80.

Experimental studies have provided compelling evidence for the crucial role of NLRC3 in regulating the cGAS-STING pathway. Cellular models have demonstrated that NLRC3 overexpression significantly inhibits cGAS-STING pathway activation, leading to a reduction in type I interferon production. Conversely, NLRC3 knockout or inhibition enhances the activity of this pathway, resulting in augmented immune responses 81, 82. These findings have been further validated by analyzing cytokine levels and the phosphorylation status of key signaling molecules 83, 84. Animal models have provided further confirmation of NLRC3's importance in immune regulation. NLRC3 knockout mice exhibit exaggerated inflammatory responses and heightened immune reactions to DNA virus infection, underscoring the essential role of NLRC3 in controlling overactive immune responses. In tumor models, NLRC3 knockout mice display enhanced anti-tumor immune responses, suggesting that NLRC3 contributes to tumor immune evasion by regulating the cGAS-STING pathway. These mice exhibit increased CD8+ T cell activity and more potent cytotoxic responses against tumor cells 85. Clinical data have also corroborated the significance of NLRC3 in immune regulation. Studies have revealed that NLRC3 expression is significantly reduced in a variety of inflammatory diseases and cancers, suggesting its potential utility as a biomarker for these conditions. Notably, alterations in NLRC3 expression are closely associated with disease progression and treatment response in patients. For instance, in lung cancer patients, low NLRC3 expression correlates with poor prognosis, implying a potential tumor-suppressive role for NLRC3 in disease progression. Analyzing NLRC3 expression levels in patient samples could provide valuable insights for personalized therapy, aiding in the prediction of treatment outcomes and monitoring disease progression 86, 87.

By further elucidating the intricate interplay between NLRC3 and the cGAS-STING pathway, scientists aim to develop novel therapeutic strategies based on NLRC3 modulation to fine-tune immune responses and improve disease treatment outcomes 88, 89. Future research will continue to investigate the functional changes that occur in NLRC3 under different pathological conditions and their impact on immune responses, with the ultimate goal of improving treatments for inflammatory diseases and cancer. These efforts are crucial for advancing personalized medicine and providing more precise and effective treatment options for patients 90, 91.

## Application prospects of NLRC3 in immunotherapy of lung cancer

NLRC3, acting as a negative regulator of the cGAS-STING pathway, holds significant promise as a therapeutic target for lung cancer immunotherapy. Inhibiting NLRC3 function has the potential to enhance cGAS-STING pathway activity, thereby bolstering anti-tumor immune responses and opening new avenues for drug development and gene therapy 92, 93. In the realm of drug development, small-molecule inhibitors and antibody drugs are being designed to specifically block NLRC3 activity, thereby activating the cGAS-STING pathway and potentiating anti-tumor immune responses 64, 94. These drugs function by binding to NLRC3, effectively inhibiting its negative regulatory function and unleashing the anti-tumor potential of the cGAS-STING pathway.

In the field of gene therapy, CRISPR-Cas9 and other cutting-edge gene editing technologies are being employed to knock out or silence the NLRC3 gene, aiming to enhance the immune system's ability to recognize and eliminate tumor cells. The precision and efficiency of gene editing technologies have shown particularly promising results in preclinical studies 95. Preclinical studies have demonstrated that inhibiting NLRC3 can significantly augment the efficacy of immune checkpoint inhibitors, leading to improved treatment outcomes in lung cancer. Notably, in animal models, combining NLRC3 inhibitors with PD-1/PD-L1 inhibitors significantly suppresses tumor growth while simultaneously increasing T cell infiltration and activity within the tumor microenvironment 21, 96. These encouraging findings provide a solid foundation for further clinical trials, and several studies are already underway to evaluate the safety and efficacy of NLRC3-targeted therapies in human patients 97, 98.

Importantly, the regulatory scope of NLRC3 extends beyond direct modulation of immune responses; it encompasses a broader immune regulatory network. NLRC3, by virtue of its influence over the cGAS-STING pathway, affects the expression of various inflammatory mediators and signaling molecules, thereby regulating immune responses at multiple levels. This multifaceted regulatory capability significantly broadens the potential applications of NLRC3 in immunotherapy 9, 99. A deeper understanding of the molecular mechanisms and functional intricacies of NLRC3 could pave the way for more precise and effective therapeutic strategies, further improving treatment outcomes and survival rates for lung cancer patients 48.

The pursuit of novel strategies to modulate the cGAS-STING pathway is a vibrant area of research, with NLRC3-based immunotherapy emerging as a particularly promising avenue. Precisely modulating NLRC3 activity holds the potential to amplify anti-tumor immune responses while simultaneously mitigating the risk of tissue damage that can arise from excessive immune reactions. Specific strategies currently under investigation include directly inhibiting NLRC3 using small-molecule inhibitors and enhancing cGAS-STING pathway activity through sophisticated gene editing technologies designed to either delete or silence the NLRC3 gene 100. Encouragingly, preclinical studies have demonstrated that NLRC3 inhibition can be synergistically combined with existing immune checkpoint inhibitors to achieve significantly improved therapeutic outcomes. For instance, studies have revealed that the combination of NLRC3 inhibitors with STING agonists can markedly enhance T cell activity and bolster their anti-tumor capabilities 82, 101. This combinatorial therapeutic approach not only augments tumor immunogenicity but also empowers the immune system to mount a more sustained and effective attack against tumor cells. Furthermore, the exploration of novel cGAS-STING pathway modulators has extended to include combinations with other immunomodulatory agents to further refine and optimize therapeutic regimens. Combining TLR agonists with NLRC3 inhibitors, for example, holds the promise of concurrently activating multiple immune pathways, thereby eliciting a more robust and comprehensive anti-tumor immune response 102, 103. Preclinical studies employing these innovative strategies have yielded compelling anti-tumor effects, establishing a solid theoretical foundation for their clinical translation. Anticipating these advancements, future clinical studies will be essential to rigorously evaluate the safety and efficacy of these combination therapies, paving the way for their successful integration into clinical practice 104, 105.

Combination therapies have emerged as a beacon of hope in the realm of lung cancer immunotherapy, offering the potential to achieve superior therapeutic outcomes and overcome the inherent limitations of single-agent therapies. The synergistic combination of NLRC3 inhibitors, cGAS-STING pathway agonists, and immune checkpoint inhibitors holds particular promise for enhancing anti-tumor immune responses on multiple fronts. However, several limitations and challenges must be addressed to fully realize the therapeutic potential of these novel approaches. These challenges include ensuring the specificity and safety of NLRC3 inhibitors and refining the precision and efficacy of gene editing technologies 82, 100, 101.

The development of clinically viable NLRC3 inhibitors necessitates meticulous attention to specificity to prevent unintended off-target effects on other critical immune regulatory pathways. Moreover, their long-term safety profiles must be rigorously assessed. While gene editing technologies hold immense promise, their clinical translation is not without its hurdles, including technical and ethical considerations surrounding off-target effects and the enduring impact of gene edits on patients 102, 104. Additionally, overcoming the technical and biological challenges inherent in effectively activating the cGAS-STING pathway without triggering excessive or detrimental inflammation and maintaining drug stability and activity within the complex and often immunosuppressive tumor microenvironment remains paramount 103, 106.

A major bottleneck in the clinical translation of these promising laboratory findings lies in successfully bridging the gap between bench and bedside, translating these discoveries into safe and effective clinical treatments. Rigorous clinical trials are essential to validate the efficacy and safety of these novel strategies in human patients 107, 108. Furthermore, optimizing drug delivery systems to ensure effective drug concentrations and sustained therapeutic activity within the tumor microenvironment remains an area of active investigation. Patient variability represents an additional layer of complexity, underscoring the urgent need for personalized therapeutic approaches tailored to individual patients' unique tumor profiles and immune responses 109, 110.

Despite these challenges, NLRC3-based immunotherapy strategies remain a beacon of hope in the fight against lung cancer. Continued research and technological advancements are anticipated to yield significant breakthroughs in this rapidly evolving field. Future research endeavors will undoubtedly require multidisciplinary collaboration to effectively address the technical and biological challenges that lie ahead and to successfully translate these promising new strategies from the laboratory to the clinic, ultimately improving the lives of lung cancer patients. By optimizing treatment regimens and developing novel combination therapies, scientists strive to provide more effective, precise, and personalized treatment options for lung cancer patients, offering renewed hope for improved outcomes and longer, healthier lives 111, 112.

## Discussion

This review has highlighted the burgeoning potential of NLRC3 as a promising target for lung cancer immunotherapy, summarizing recent research progress in this exciting field. As a critical negative regulator of the cGAS-STING pathway, NLRC3 exerts significant influence over the magnitude and efficacy of immune responses. Compelling research has demonstrated that inhibiting NLRC3 function unleashes the anti-tumor potential of the cGAS-STING pathway, leading to enhanced anti-tumor immune responses and highlighting its therapeutic promise 92.

In the realm of drug development, both small-molecule inhibitors and gene editing technologies have shown encouraging results in preclinical studies. Small-molecule inhibitors function by directly blocking NLRC3 activity, thereby promoting cGAS-STING pathway activation, while gene editing technologies aim to enhance immune system responses by either knocking out or silencing the NLRC3 gene 100. Preclinical studies have provided compelling evidence that NLRC3 inhibition can significantly augment the efficacy of immune checkpoint inhibitors, leading to improved treatment outcomes in preclinical models of lung cancer. For example, the combination of NLRC3 inhibitors with PD-1/PD-L1 inhibitors has been shown to not only suppress tumor growth but also to increase T cell infiltration and activity within the tumor microenvironment, underscoring the potent anti-tumor effects of this combinatorial approach 113. Importantly, NLRC3's regulatory reach extends beyond direct modulation of the cGAS-STING pathway; it encompasses a broader immune regulatory network, influencing the expression of a diverse array of inflammatory mediators and signaling molecules, and thereby fine-tuning immune responses at multiple levels. This highlights the multifaceted nature of NLRC3 and its profound importance in shaping anti-tumor immunity in lung cancer.

Future research endeavors should prioritize elucidating the intricate mechanisms by which NLRC3 regulates the cGAS-STING pathway in lung cancer and leverage this knowledge to optimize therapeutic strategies. Basic research is essential to deepen our understanding of the precise molecular mechanisms governing NLRC3-mediated regulation of the cGAS-STING pathway, unraveling more specific regulatory networks and signaling pathways. Concurrently, clinical studies are critical to evaluate the safety and efficacy of NLRC3-based therapies in human patients and to explore personalized treatment approaches tailored to individual patients' tumor and immune profiles.

Promising new research avenues include investigating the role of NLRC3 in other cancer types, exploring novel regulatory mechanisms and potential interactions with other immune modulators, and developing innovative therapeutic strategies, such as next-generation combination therapies and more targeted drug delivery systems, to further enhance treatment efficacy. Through sustained research efforts and continued innovation, scientists strive to translate the promise of NLRC3-based immunotherapy into tangible clinical benefits for lung cancer patients, ultimately improving patient outcomes and advancing the field.
